# SOD Enzymes and Microbial Pathogens: Surviving the Oxidative Storm of Infection

**DOI:** 10.1371/journal.ppat.1005295

**Published:** 2016-01-07

**Authors:** Chynna N. Broxton, Valeria C. Culotta

**Affiliations:** Department of Biochemistry and Molecular Biology, Johns Hopkins University Bloomberg School of Public Health, Baltimore, Maryland, United States of America; McGill University, CANADA

## Living with ROS through Superoxide Dismutase Enzymes

Since oxygen appeared in the biosphere some 3–5 billion years ago, all organisms have had to deal with the hazards of potentially damaging reactive oxygen species (ROS), such as superoxide, hydrogen peroxide, and hydroxyl radical. Like all organisms, pathogenic microbes produce ROS as byproducts of aerobic metabolism, but the burden of ROS is magnified when these microbes confront the oxidative burst of the host. As part of the innate immune response, macrophages and neutrophils attack invading microbes with toxic superoxide [1]. To counteract this attack, some microbial pathogens express superoxide dismutase enzymes (SOD).

**Fig 1:**
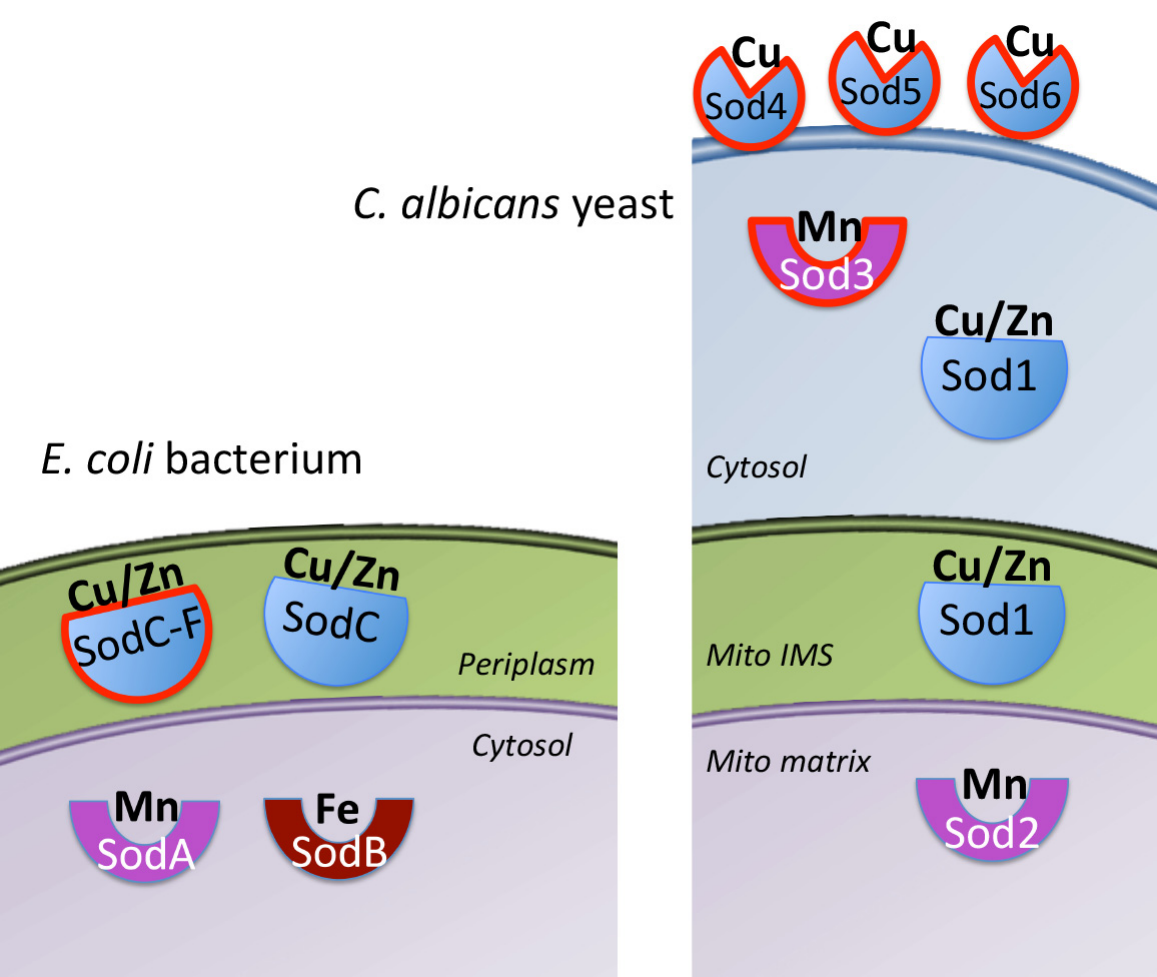
The family of SOD enzymes in microbial pathogens. In a gram-negative bacteria such as *E*. *coli* (left), the Mn-SodA and Fe-SodA are intracellular/cytosolic where Mn and Fe metals ions are generally bioavailable. Cu is extruded into the periplasmic space, driving the evolution of Cu/Zn-containing SodC in this extra-cytosolic compartment. In eukaryotes (right), the mitochondria thought to evolve from a gram-negative bacteria exhibits the equivalent partitioning of a Mn-Sod2 to the mitochondrial matrix and Cu/Zn Sod1 to the mitochondrial IMS and cytosol. Some pathogenic microbes have acquired additional SODs (lined in red), including the highly stable prophage-derived SodC-F in pathogenic *E*. *coli* (left) and in *Candida albicans* (right), the cytosolic Mn-Sod3 as a backup for Cu/Zn Sod1, and the Cu-only extracellular Sod4, Sod5, and Sod6.

SODs are metalloenzymes that use a redox-active metal to disproportionate two molecules of superoxide to oxygen and hydrogen peroxide, the latter of which is removed by catalase and peroxidase enzymes. SODs have evolved on three separate occasions, yielding a family of Mn and Fe SODs (that use either metal as co-factor), a Cu/Zn SOD family that uses Cu for catalysis, and a rare family of Ni SODs [2]. Why so many SODs? This is best answered in terms of metal bioavailability. In a typical gram-negative bacteria, such as *Escherichia coli*, the cytosol can have ample Mn and/or Fe, but Cu is extruded into the periplasmic/extracellular space [3]. As a result, Mn and Fe SODs are generally intracellular/cytosolic while Cu/Zn SODs are extracellular/periplasmic (Fig 1). Consistent with the endosymbiosis theory of mitochondrial evolution, this partitioning of SOD enzymes has been retained in eukaryotic mitochondria: The mitochondrial matrix (equivalent to bacterial cytosol) harbors a Mn SOD, while Cu/Zn SOD is in the mitochondrial intermembrane space and cytosol (equivalent to bacterial periplasmic/extracellular) (Fig 1).

## Custom SOD Enzymes for Bacterial Pathogenesis

The accepted nomenclature for bacterial SODs is SodA, SodB, and SodC for the Mn, Fe, and Cu/Zn SODs, respectively. Because superoxide does not generally cross biological membranes, the intracellular SodA and SodB largely remove intracellular or metabolic sources of superoxide while the periplasmic/extracellular SodC directly combats superoxide from the animal host. Not all bacterial pathogens contain extracellular SodC; for example, the Lyme disease pathogen *Borrelia burgdorferi* contains a single Mn-SodA. This pathogen also does not express Fe-SodB, representing a clever adaptation to limiting Fe supplies in the host [4,5].

The roles of SodA and SodB in bacterial survival and pathogenesis vary greatly depending on the species. In some cases (e.g., *Salmonella typhimurium*), loss of SodA has no impact on virulence [6], whereas in other instances (e.g., *Streptococcus agalactiae* or *B*. *burgdorferi*), loss of the intracellular SOD attenuates virulence, in spite of there being no growth inhibition in laboratory cultures [7,8]. How could an intracellular SOD promote virulence when host superoxide is extracellular? Although superoxide does not typically cross biological membranes, it can do so when protonated. In low pH environments, such as that of a macrophage phagolysosome, the protonated superoxide might enter the bacteria and serve as substrate for intracellular SodA and SodB [9]. Alternatively, the intracellular SODs may exclusively prevent damage from bacteria-derived superoxide that somehow promotes microbial fitness during infection.

Compared to SodA and SodB, the extracellular Cu/Zn SodC is well known to react with host superoxide and is a documented virulence factor for many bacteria [10]. In certain microbes, nature seems to have improved on the Cu/Zn SOD template to support pathogenesis. SodC was originally identified by Ludmil T. Benov and Irwin Fridovich in non-pathogenic laboratory strains of *E*. *coli* [11], but pathogenic *E*. *coli* including the highly virulent enteropathogenic O157:H7 serotype has acquired additional SodC genes through horizontal prophage gene transfer (Fig 1). This so-called SodC-F appears superior to chromosomal SodC in terms of protease resistance and enhanced stability for Cu binding [12]. Interestingly, this same pattern is seen with *Salmonella*, where all strains have a chromosomal SodCII, but highly pathogenic serotypes also carry prophage-derived SodC1 that is resistant to degradation by host proteases and exhibits superior Cu binding stability [13]. The prophage SodCII is more crucial for *Salmonella* virulence than the chromosomal SodC [14]. The enhanced biochemical stability of these prophage-derived SODs should prove advantageous to the pathogen in the harsh environment of the host.

Up until 2004, all Cu-containing SODs were believed to also contain a Zn co-factor. Zn does not participate directly in enzyme catalysis, but stabilizes the polypeptide and fine-tunes the redox properties of the catalytic Cu. Surprisingly, SodC of *Mycobacterium tuberculosis* and of the closely related *M*. *leprae* and *M*. *avian* species is an enzymatically active Cu-SOD that lacks Zn [15]. This Cu-only SOD is well suited to function under Zn-limited conditions. As part of a nutritional immunity response, the host attempts to starve pathogens of nutrient Zn [16], which would have no consequence on a Cu-only SOD.

## Fungal Adaptations to Pathogenesis through Specialized SOD Enzymes

As with other eukaryotes, pathogenic fungi express a largely cytosolic Cu/Zn Sod1 and a distinct Mn-containing Sod2 in the mitochondrial matrix (Fig 1). Cu/Zn Sod1 is a documented virulence factor for *Cryptococcus neoformans* and *Candida albicans* [17,18]. Activity of fungal Sod1 is limited by the availability of its Cu co-factor [19], and Cu inside the host can vary tremendously. Cu can become very high in activated macrophages [20], and consistent with this, *C*. *albicans* mutants defective in Cu detoxification show impairments in macrophage invasion [21]. Cu can also become high in specific host niches, such as in lungs infected with *C*. *neoformans* and in the bloodstream during *C*. *albicans* and *C*. *neoformans* invasion [22,23]. However, in tissues that are targeted by *C*. *neoformans* and *C*. *albicans* (such as brain and kidney tissues), Cu availability can become very low [23,24]. We have recently shown that *C*. *albicans* adapts to such variations in host Cu by adjusting its metal co-factor selection for SODs. When host Cu is high, the yeast expresses Cu/Zn Sod1, but when host Cu is low, *C*. *albicans* will switch to a non-Cu alternative, namely a cytosolic Mn-Sod3 (Fig 1) [23]. Cytosolic Mn SODs are extremely rare in biology, and the unusual expression of Mn-Sod3 in the *C*. *albicans* cytosol endows this pathogen with uninterrupted SOD activity irrespective of host Cu [23].

**Fig 2:**
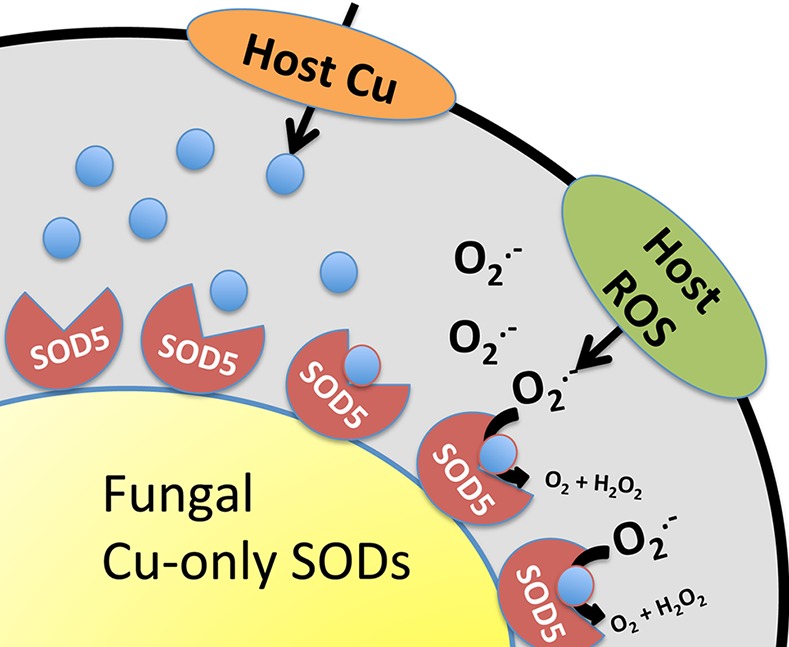
Model for Cu-only fungal SOD5 at the macrophage–pathogen interface. Activated macrophages may attack invading microbes with toxic levels of Cu and superoxide (O_2_^-^). With its open Cu site, Sod5 on the surface of the fungal pathogen may be able to capture this host Cu, fueling the SOD enzyme to remove the superoxide in counterattack.

In addition to this adaptation with intracellular SODs, certain fungal pathogens possess unusual extracellular Cu/Zn-like SODs that appear tailor-made for host invasion. In *C*. *albicans*, three extracellular Cu/Zn-like SODs (Sod4, Sod5, and Sod6) are attached to the cell wall through glycosylphosphatidylinositol (GPI) anchors (Fig 1). Of these, Sod5 has been well characterized and is known to directly remove host cell-derived superoxide and to promote virulence in animal models [25,26]. Upon close inspection, we noted that Sod4, Sod5, and Sod6 can only bind Cu (not Zn), making these SODs akin to Cu-only SodC of *M*. *tuberculosis* [15,27]. However, unlike Cu-only SodC, the fungal SODs also lack a region of the SOD protein known as the “electrostatic loop” (ESL), named for its role in electrostatically guiding superoxide to the active site [28]. In spite of having no ESL and no Zn, *C*. *albicans* Sod5 is a very active SOD [27]. With no ESL, the Cu site of Sod5 is surface-exposed, as opposed to the buried Cu ion of Cu/Zn SODs and *Mycobacterium* Cu-only SodC [27]. We propose that this open Cu site allows the fungal SOD to avidly capture Cu from the host (Fig 2). In particular niches, host Cu can become very high, and Sod5-like SODs may use this Cu to mount a powerful defense against the superoxide attack (Fig 2). Sod5-like SODs (Cu-only, no ESL, extracellular GPI-anchored) can be found throughout Basidiomycota and Ascomycota fungi [27], and they are essential for virulence of other fungi, including the pulmonary pathogen *Histoplasma capsulatum* [29]. Not all fungal pathogens express extracellular Cu-SODs; an example is *C*. *neoformans*. Additionally, there are examples of fungi predicted to express Sod5-like SODs that are not established pathogens, such as *Podospora anserine* [27]. Interestingly, *P*. *anserine* produces ROS as part of a differentiation process [30] and may employ its Sod5-like SOD to handle fungal-derived, rather than host-derived, superoxide.

## Concluding Remarks

While all aerobic organisms express SODs for endogenous superoxide, many pathogens have been armed with additional SODs designed to function in the hostile climate of the host–pathogen interface. This is particularly true for extracellular Cu-SODs of bacteria and fungi that lie in the direct line of fire from host superoxide. The phage-acquired SodCs of pathogenic *E*. *coli* and *Salmonella* are resilient towards host proteases and will not readily surrender their Cu co-factor to the host. Additionally, the Cu-only SODs of *Mycobacterium* and of fungal pathogens appear optimally designed to function in host environments of low Zn and high Cu. As a final thought, it is worth mentioning that the host cell itself must endure the oxidative insult of its own doing. Like the invading microbe, host cells secrete Cu/Zn SODs to manage extracellular superoxide, but how well this host SOD has evolved to endure the infection battleground remains to be determined.
